# The impact on incident tuberculosis by kidney function impairment status: analysis of severity relationship

**DOI:** 10.1186/s12931-020-1294-5

**Published:** 2020-02-12

**Authors:** Chin-Chung Shu, Yu-Feng Wei, Yi-Chun Yeh, Hsien-Ho Lin, Chung-Yu Chen, Ping-Huai Wang, Shih-Lung Cheng, Jann-Yuan Wang, Chong-Jen Yu

**Affiliations:** 1 Department of Internal Medicine, National Taiwan University Hospital, NO 7, Chung-Shan South Road, Taipei, Taiwan 100 Taiwan; 2 College of Medicine, National Taiwan University, Taipei, Taiwan; 3 School of Medicine for International Students, College of Medicine, I-Shou University, Kaohsiung, Taiwan; 4 Division of Chest Medicine, Department of Internal Medicine, E-Da Hospital, Kaohsiung, Taiwan; 5 Department of Medical Research, National Taiwan University Hospital, Taipei, Taiwan; 6 Institute of Epidemiology and Preventive Medicine, College of Public Health, National Taiwan University, Taipei, Taiwan; 7 Department of Internal Medicine, National Taiwan University Hospital Yunlin Branch, Yunlin County, Taiwan; 8 Department of Internal Medicine, Far-Eastern Memorial Hospital, New Taipei, city, Taiwan; 9 Department of Chemical Engineering and Materials Science, Yuan Ze University, Zhongli City, Taoyuan County Taiwan

**Keywords:** Tuberculosis, Kidney function, Chronic kidney disease, Dialysis, Risk

## Abstract

**Background:**

The risk of tuberculosis (TB) in patients with impaired kidney function remains unclear by different stages of renal function impairment.

**Methods:**

We retrospectively recruited all patients with kidney function in a tertiary-care referral center from January 2008 to December 2013 and followed them till December 2016. We defined the primary outcome as active TB development and analyzed the impact of kidney function impairment.

**Results:**

During the study period, a total of 289,579 patients were enrolled for analysis, and of them, 1012 patients had active TB events in an average of 4.13 years of follow-up. According to kidney function impairment, the incidence rate of TB was similar in patients with no chronic kidney disease (CKD) or stage 1 and stage 2, and it increased apparently at stage 3a (167.68 per 100,000 person-years) to stage 3b, stage 4 and stage 5 (229.25, 304.95 and 349.29 per 100,000 person-years, respectively). In a Cox proportional hazard regression model, the dose response of TB risk among different stages of kidney function impairment increased significantly from CKD stage 3a to stage 5. Patients with long-term dialysis had a hazard ratio of 2.041 (1.092–3.815, *p* = 0.0254), which is similar to that of stage 4 CKD but lower than that of stage 5.

**Conclusion:**

In patients with impaired kidney function, the risk of TB increases from CKD stage 3, and in stage 5, the risk is even higher than that of those receiving dialysis. Further strategies of TB control need to consider this high-risk group.

## Background

According to the World Health Organization (WHO), tuberculosis (TB) remains the most common infectious disease in the world [1]. In 2017, an estimated 10.0 million people had active TB, and 1.3 million TB related deaths were recorded worldwide [2]. In many countries, TB has declined from high incidence (> 100/100000 person-years) to intermediate (30–100/100000) or low incidence (< 30/100000) [3, 4] under the Global Plan To Stop TB 2006–2015 [5]. In the post-2015 era, WHO suggest the END TB strategy and many frameworks towards TB elimination [6], including to optimize current treatment and to screen high-risk groups for latent TB infection (LTBI) treatment [7]. The WHO suggests a focus on high-risk groups in high or upper-middle income countries when the TB incidence becomes less than 100 per 100,000 person-years [8].

Of the high-risk groups, patients with end-stage renal disease need long-term dialysis is highly recommended for LTBI screening [7], but the risk of TB in patients with decreased kidney function, such as chronic kidney disease (CKD) not requiring dialysis, is still unclear. In particular, the burden of CKD is increasing worldwide, so its correlation with infection of TB is increasing in importance [9]. Actually, immune deficiency due to decline in kidney function is a risk factor for infection [10] and has reportedly been correlated with infection associated mortality [11, 12]. Due to changes in the immune system due to deterioration of kidney function, active TB is an infectious complication that may develop or reactivate from LTBI.

In fact, few studies have examined TB risk in patients with kidney function impairment [13]. Only a previous cohort study reports that an adjusted hazard ratio of pulmonary TB was 1.45-fold higher in the CKD group than the non-CKD group [14]. But there is no study shows the TB risk according to the different stages of kidney function impairment [13], therefore, we cannot pick out which sub-population with kidney function impairment for implementation of LTBI strategy. Hence, we conducted this retrospective cohort study to analyze the correlation between kidney function and the incidence of TB in Taiwan, an intermediate TB country with incidence of 38.9 per 100,000 person-years in 2018 [15], using a large hospital-based cohort.

## Methods

### Participant enrollment

This retrospective study was conducted in a tertiary-care referral center in northern Taiwan under the approval of the Institutional Review Board of Research Ethics Committee of National Taiwan University Hospital (NO. 201510009RINB). From January 2008 to December 2013, patients aged ≥20 years were identified. We included patients with data of serum creatinine and excluded those having follow-up periods at the study hospital of less than 6 months and 3 months, respectively, before and after the index data of initial kidney function. In addition, we excluded those who had active tuberculosis before recruitment and within 3 months right after the index data of the kidney function.

### Definitions of kidney function, active tuberculosis and co-morbidity

We retrieved the participants’ clinical information such as age, gender, body mass index, and underlying disease from the hospital’s electronic record database within a total 12 months (6 months before and after the index date). We used an abbreviated Modification of Diet in Renal Disease (MDRD) equation to estimate the glomerular filtration rate (eGFR) [16] and classified the patient’s initial kidney function according to the guidelines of the National Kidney Foundation [17]. The eGFR of ≥90, < 90~ ≥ 60, < 60~ ≥ 45, < 45~ ≥ 30, < 30~ ≥ 15, and < 15 ml/min/1.73m^2^ stood for no CKD or stage 1, stage 2, stage 3a, stage 3b, stage 4, and stage 5, respectively. We defined active TB by positive culture result for *Mycobacterium tuberculosis*. For the diagnosis without microbiological evidence, we could define active TB by the unique diagnosis code in electronic chart record, because active TB is a certifiable infectious disease that needs to report to the Taiwan Centers for Disease Control. We used one time of admission diagnosis, or at least two outpatient clinic diagnoses plus prescription of anti-tuberculous agents for TB diagnosis. Co-morbidity of long-term dialysis, transplantation, pneumoconiosis, idiopathic pulmonary fibrosis, cirrhosis of the liver, cancer, lupus erythematous, rheumatoid arthritis, polymyositis, and dermatomyositis were defined by a record of a catastrophic illness card. Other underlying diseases were categorized by one admission diagnosis or more than 2 outpatient clinic diagnoses.

### Outcome and statistical analysis

We followed up all enrolled participants till December 2016 and defined occurrence of active TB as the primary outcome. The incidence of TB was calculated as the average number per 100,000 person-years. Inter-group differences were compared using *t* test or one-way ANOVA for continuous variables, where appropriate, and *chi* squared test for categorical variables. We used Cox proportional hazard regression for time to TB event analysis. Once the patients with CKD at the initial received long-term dialysis or kidney transplantation in the follow-up or were lost to follow-up, they were censored in the time dependent analysis. We combined no CKD or stage 1 and stage 2 as a reference group (≥ 60 min/ml per 1.73m^2^) in Cox regression analysis because their TB incidence were similar [18]. We calculated the correlations between the kidney function of eGFR and the univariate hazard ratio for TB by Spline Cox proportional regression. We investigated the risk factors for active TB using multivariate Cox proportional hazard regression analysis with possible associated variables from univariate analysis. Only variables with a two-sided *p* < 0.05 were retained in the final model. All analyses were performed in SAS version 9.4 (SAS Institute Inc., Cary, NC, USA).

## Results

### Demographics of patients with different stages of kidney function impairment

**Fig. 1:**
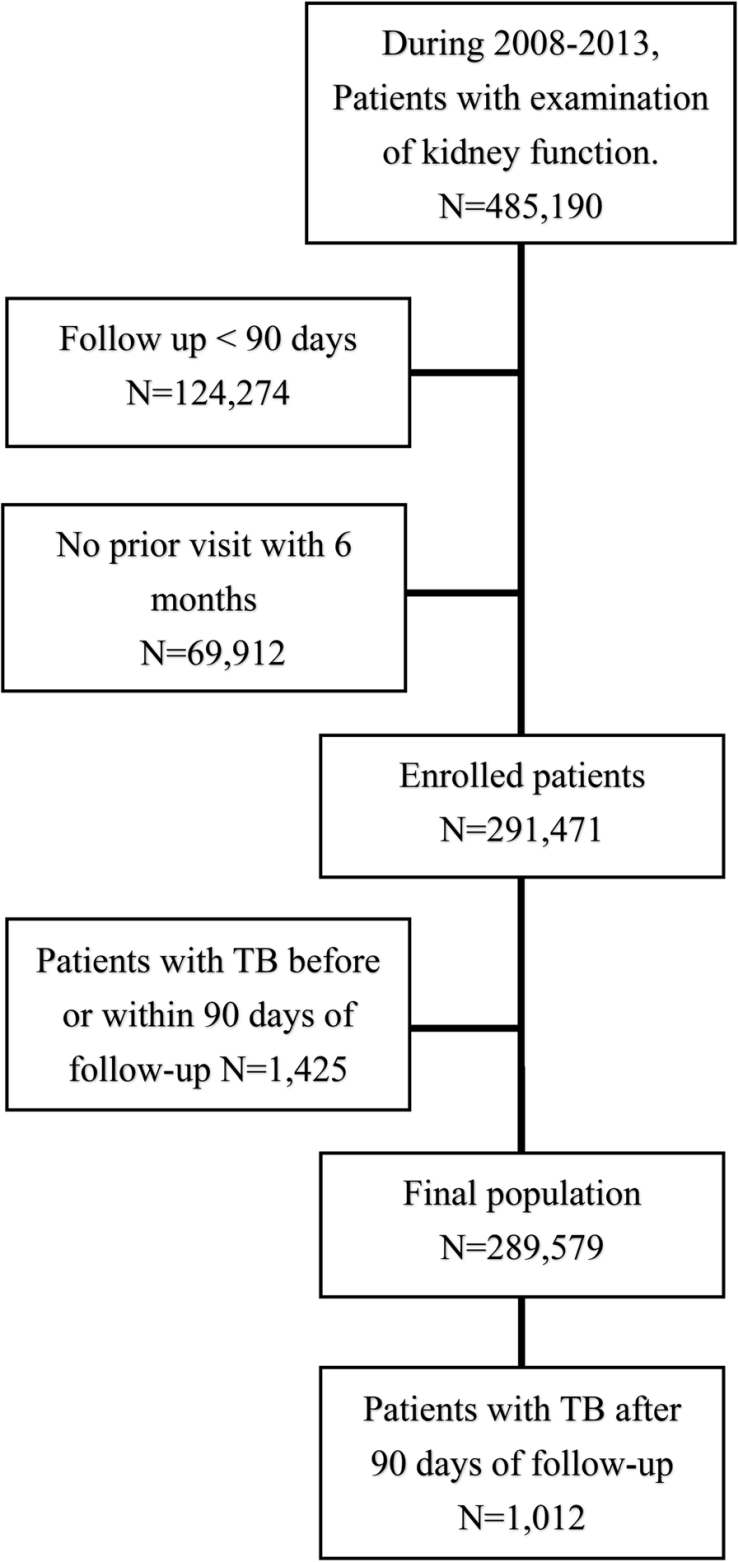
Flow chart of study. TB, tuberculosis

During the study period, a total of 485,190 patients in the study hospital were eligible, and 289,579 were enrolled for analysis after excluding 124,274 for short follow-up (< 90 days), 69,912 for short previous data (< 6 months) and 1425 for having TB within 90 days (Fig. 1). Among them, 1012 patients had development of active TB events during the overall follow up of 1,196,206 person-years, that was averagely 4.13 years/person. Among those with different stages of kidney function impairment (Table 1), old age and male predominance were generally found for those with advanced CKD stages. The mean age of all participants was 5.18 years old (SD: 17.3). The mean age was 43.8 (15.2) in no CKD or stage 1, 55.5 (15.7) in stage 2, 69.5 (12.4) in stage 3a, 71.6 (12.8) in stage 3b, 70.1 (14.2) in stage 4, 62.7 (15.7) in stage 5, 56.9 (14.4) in dialysis, and 54.8 (11.7) in transplant group (*p* < 0.0001, Table 1). Male gender was around 45.3% in all participants and distributed as 39.7, 49.4, 51.9, 50.3, 50.4, and 52.2% from no CKD or stage 1, stage 2, 3a, 3b, 4 and 5, respectively. Many underlying comorbidities significantly increased in advanced CKD stage included CHF, stroke, DM and SLE. By contrast, obesity was less frequently in in patients with worse kidney function than those with no CKD or stage 1 and 2.

**Table 1:** Demographics of study population according to kidney function

Variables	CKD						Long term dialysis (*n* = 1579)	Kidney transplant (*n* = 38)	*P*-value
	nil or stage 1 (*n* = 129,421)	2 (*n* = 128,248)	3a (*n* = 17,228)	3b (*n* = 7116)	4 (*n* = 3449)	5 (*n* = 2500)			
Age (years)	43.8 ± 15.2	55.5 ± 15.7	69.5 ± 12.4	71.6 ± 12.8	70.1 ± 14.2	62.7 ± 15.1	56.9 ± 14.4	54.8 ± 11.7	< 0.0001
Age group									< 0.0001
≤ 40	57,461 (44.4)	23,076 (18.0)	409 (2.4)	151 (2.1)	141 (4.1)	226 (9.0)	204 (12.9)	5 (13.2)	
40–55	38,907 (30.1)	35,828 (27.9)	1744 (10.1)	613 (8.6)	380 (11.0)	514 (20.6)	499 (31.6)	12 (31.6)	
55–65	21,780 (16.8)	31,925 (24.9)	3430 (19.9)	1175 (16.5)	537 (15.6)	586 (23.4)	404 (25.6)	14 (36.8)	
> 65	11,273 (8.7)	37,419 (29.2)	11,645 (67.6)	5177 (72.8)	2391 (69.3)	1174 (47.0)	472 (29.9)	7 (18.4)	
Male gender	51,354 (39.7)	63,394 (49.4)	8942 (51.9)	3582 (50.3)	1738 (50.4)	1304 (52.2)	804 (50.9)	27 (71.1)	< 0.0001
Serum Creatinine	0.7 ± 0.1	0.9 ± 0.2	1.2 ± 0.2	1.7 ± 0.3	2.6 ± 0.6	6.8 ± 3.0	7.2 ± 3.2	2.6 ± 2.2	<0.0001
eGFR	108.2 ± 18.2	77.6 ± 8.0	53.8 ± 4.2	38.5 ± 4.3	23.3 ± 4.3	9.0 ± 3.3	11.4 ± 12.9	47.9 ± 31.9	< 0.0001
COPD	767 (0.6)	1512 (1.2)	491 (2.9)	231 (3.3)	92 (2.7)	44 (1.8)	10 (0.6)	1 (2.6)	< 0.0001
Asthma	1320 (1.0)	1566 (1.2)	285 (1.7)	117 (1.6)	35 (1.0)	20 (0.8)	11 (0.7)	0 (0.0)	< 0.0001
Bronchiectasis	389 (0.3)	415 (0.3)	53 (0.3)	24 (0.3)	12 (0.4)	5 (0.2)	0 (0.0)	0 (0.0)	0.3808
Pneumoconiosis	34 (0.0)	48 (0.0)	13 (0.1)	6 (0.1)	1 (0.0)	0 (0.0)	3 (0.2)	0 (0.0)	NA
IPF	182 (0.1)	178 (0.1)	32 (0.2)	10 (0.1)	9 (0.3)	6 (0.2)	1 (0.1)	0 (0.0)	NA
GERD	4119 (3.2)	5036 (3.9)	630 (3.7)	228 (3.2)	101 (2.9)	79 (3.2)	47 (3.0)	0 (0.0)	< 0.0001
Obesity	891 (0.7)	685 (0.5)	40 (0.2)	13 (0.2)	14 (0.4)	7 (0.3)	1 (0.1)	0 (0.0)	< 0.0001
Cancer	14,895 (11.5)	16,417 (12.8)	2964 (17.2)	1382 (19.4)	645 (18.7)	270 (10.8)	197 (12.5)	5 (13.2)	< 0.0001
Cirrhosis	1314 (1.0)	1509 (1.2)	400 (2.3)	236 (3.3)	132 (3.8)	46 (1.8)	37 (2.3)	1 (2.6)	< 0.0001
CHF	662 (0.5)	1543 (1.2)	709 (4.1)	501 (7.0)	348 (10.1)	231 (9.2)	96 (6.1)	1 (2.6)	< 0.0001
Stroke	763 (0.6)	1381 (1.1)	429 (2.5)	221 (3.1)	122 (3.5)	55 (2.2)	16 (1.0)	1 (2.6)	< 0.0001
Diabetes mellitus	8179 (6.3)	12,865 (10.0)	3771 (21.9)	2217 (31.2)	1274 (36.9)	961 (38.4)	301 (19.1)	8 (21.1)	< 0.0001
SLE	738 (0.6)	409 (0.3)	58 (0.3)	29 (0.4)	27 (0.8)	31 (1.2)	23 (1.5)	0 (0.0)	< 0.0001
RA	802 (0.6)	674 (0.5)	94 (0.6)	41 (0.6)	22 (0.6)	15 (0.6)	8 (0.5)	0 (0.0)	0.1435
Polymyositis	70 (0.1)	28 (0.0)	3 (0.0)	2 (0.0)	2 (0.1)	1 (0.0)	0 (0.0)	0 (0.0)	NA
Dermatomyositis	73 (0.1)	47 (0.0)	1 (0.0)	1 (0.0)	2 (0.1)	0 (0.0)	0 (0.0)	0 (0.0)	NA
Transplant, others	130 (0.1)	204 (0.2)	86 (0.5)	41 (0.6)	17 (0.5)	6 (0.2)	6 (0.4)	2 (5.3)	< 0.0001

Abbreviation: *BMI* Body mass index, *CHF* Congestive heart failure, *CKD* Chronic kidney disease, *COPD* Chronic obstructive pulmonary disease, *eGFR* Estimated glomerular filtration rate, *GERD* Gastroesophageal reflux disease, *IPF* Idiopathic pulmonary fibrosis, *RA* Rheumatoid arthritis, *SLE* Systemic lupus erythematosus

The number means mean ± standard deviation and number (%)

Chi-square tests for categorical variables; ANOVA for continuous variables

In comparison with patients without TB (Additional file 1: Table S1), those who developed TB during follow-up were older and predominantly male. In addition, they had lower eGFR and more advanced renal disease, dialysis, and kidney transplant. The proportion of patients with active TB development was lower in no CKD or stage 1 and 2, but it became higher in advanced CKD stage 3 to 5 in comparing with those without TB (*p* < 0.0001). The underlying diseases of pulmonary disease, gastroesophageal reflux disease, cancer, heart failure, cirrhosis, diabetes mellitus and autoimmune disease were higher in patients with TB development. By contrast, obesity was higher in patients without TB development.

### TB incidence for different kidney functions

According to kidney function, the incidence rate of TB was similar in no CKD or stage 1 and stage 2 (70.06 and 71.08 per 100,000 person-years, respectively), and increased apparently from stage 3a (167.68 per 100,000 person-years, Hazard ratio [HR]: 2.404 [1.956–2.956], *p* < 0.0001) to stage 3b, stage 4 and stage 5 (incidence: 229.25, 304.95 and 349.29 per 100,000 person-years, and HR: 3.273 [2.509–4.270], 4.317 [3.078–6.054], and 4.852 [3.154–7.646], respectively) (Table 2). Those with long-term dialysis or kidney transplant before case enrollment had TB incidences of 175.60 and 1429.33 per 100,000 person-years, respectively and HR of 2.493 [1.330–4.673] and 20.575 [5.147–82.239], respectively.

**Table 2:** Incidence rates of tuberculosis (TB) (*N* = 289,579, TB events = 1012)

Variable	TB events	N	Follow up (years/person)	Follow up (person-years)	Incidence rate (per 100,000 person-years)	Hazard Ratio	(95%CI)		*P*-value
Kidney function									
CKD nil or stage 1	362	129421	3.99	516,705.16	70.06	Reference			
CKD stage 2	395	128248	4.33	555,749.96	71.08	1.024	(0.888,	1.182)	0.7408
CKD stage 3a	120	17228	4.15	71,566.72	167.68	2.404	(1.956,	2.956)	< 0.0001
CKD stage 3b	64	7116	3.92	27,917.47	229.25	3.273	(2.509,	4.270)	< 0.0001
CKD stage 4	37	3449	3.52	12,133.22	304.95	4.317	(3.078,	6.054)	< 0.0001
CKD stage 5	22	2500	2.52	6298.52	349.29	4.852	(3.154,	7.464)	< 0.0001
Long-term dialysis	10	1579	3.61	5694.64	175.60	2.493	(1.330,	4.673)	0.0044
Kidney transplant	2	38	3.68	139.93	1429.33	20.575	(5.147,	82.239)	< 0.0001

Abbreviation: *CKD* Chronic kidney disease

For confirming that the case exclusion criteria of follow-up period ≤90 days did not influence the results of TB incidence, we used different follow-up period as exclusion criteria, including ≤30 days or ≤ 180 days and analyzed the TB incidence by different kidney function status. We found that the trends of TB incidence in the different CKD subgroups were similar (Additional file 1: Table S2 and S3).

### Risk factors and the hazard ratio for TB development

**Fig. 2:**
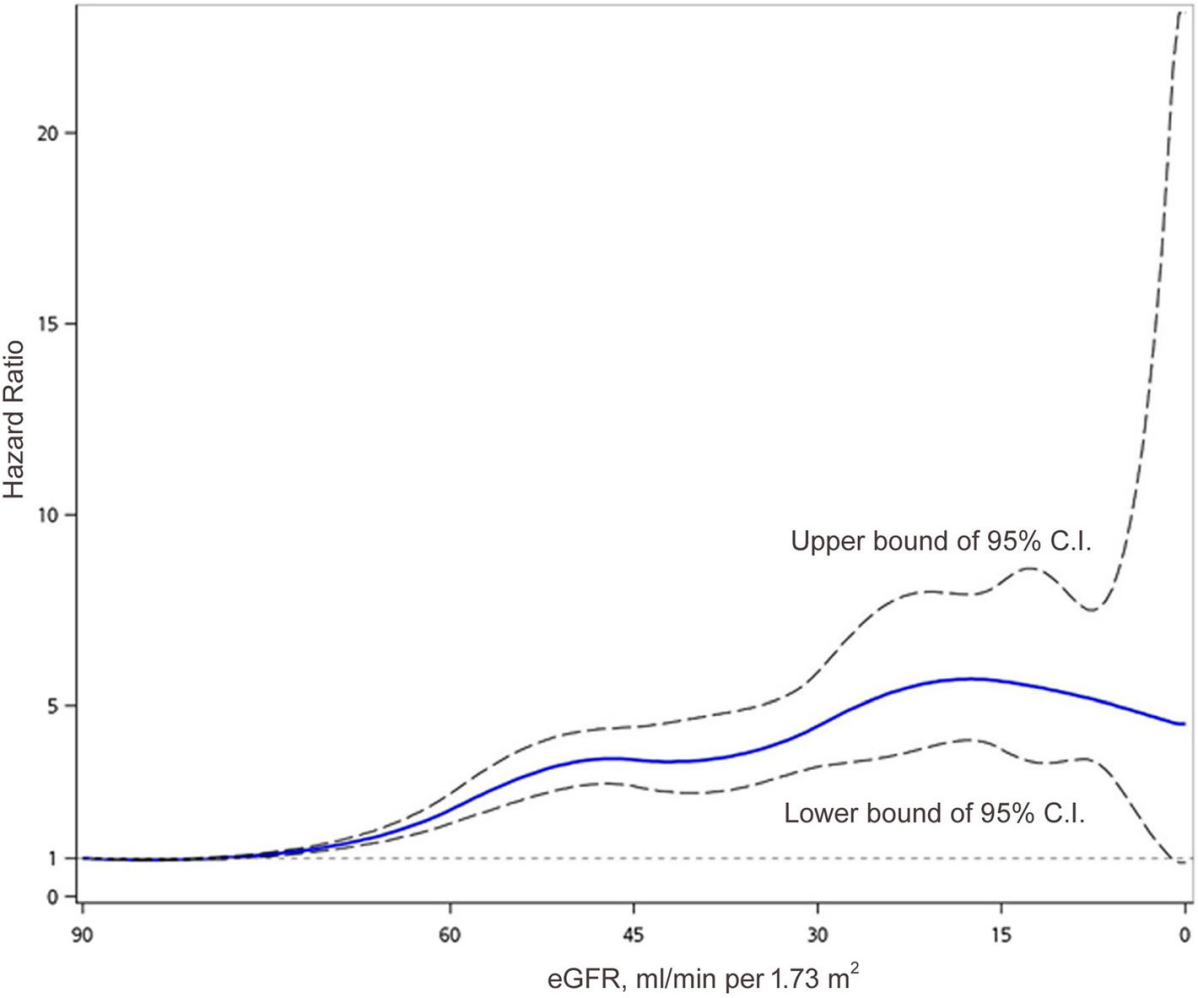
Unadjusted hazard ratio (HR) for tuberculosis development according the estimated glomerular filtration rate (eGFR, ml/min per 1.73 m^2^). Spline Cox proportional hazard regression is used for this analysis. The central curve is unadjusted HR and the upper and lower curves are the 95% C.I.

In a Cox proportional hazard regression model (Table 3), we analyzed the hazard ratio (HR) of TB development for kidney function and other clinically relevant factors. In univariate analysis, patients had similar TB risk in no CKD or stages 1 vs stage 2 (HR: 1.024 [95% C.I.: 0.888–1.182], *p* = 0.7408, compared with no CKD or stage 1), so we used them as the reference group for those with deteriorated kidney function in Cox regression analysis. In univariate analysis, the dose response of TB risk among different kidney functions increased significantly from CKD stage 3a to stage 5. When we evaluated the univariate HR of TB risk at every eGFR below 90 ml/min/1.73m^2^ using spline Cox regression, the correlation actually increased slowly in stage 2 and obviously from stage 3a. A plateau was noticed around the beginning of stage 3b and a mild decline at late stage 5. However, the outcome number was small in stage 5, and the 95% confidence interval became large (Fig. 2).

**Table 3:** Univariate and multivariate Cox proportional hazard regression analysis for development of tuberculosis

Variables	HR	95% CI		*P*-value	Adjusted HR	95% CI		*P*-value
Age, years (ref: ≤ 40)								
40–55	1.581	1.227	2.037	0.0004	1.392	1.079	1.797	**0.0109**
55–65	2.521	1.975	3.218	< 0.0001	2.040	1.592	2.614	**< 0.0001**
> 65	6.240	5.018	7.758	< 0.0001	4.439	3.530	5.583	**< 0.0001**
Male (ref: female)	2.460	2.159	2.802	< 0.0001	2.258	1.977	2.578	**< 0.0001**
Kidney function								
CKD stage1 & 2	1	–	–	–	1	–	–	–
CKD stage 3a	2.374	1.958	2.879	< 0.0001	1.215	0.995	1.484	0.0563
CKD stage 3b	3.232	2.505	4.172	< 0.0001	1.538	1.182	2.002	**0.0014**
CKD stage 4	4.263	3.065	5.930	< 0.0001	2.065	1.473	2.894	**< 0.0001**
CKD stage 5	4.792	3.135	7.325	< 0.0001	2.877	1.872	4.421	**< 0.0001**
Long-term dialysis	2.462	1.319	4.595	0.0046	2.041	1.092	3.815	**0.0254**
Kidney transplant	20.311	5.090	81.050	< 0.0001	15.269	3.806	61.252	**0.0001**
COPD	4.271	3.147	5.797	< 0.0001	1.581	1.149	2.174	**0.0049**
Asthma	1.574	1.000	2.479	0.0501				
Bronchiectasis	4.617	2.726	7.822	< 0.0001	2.959	1.717	5.098	**< 0.0001**
Pneumoconiosis	31.004	16.083	59.768	< 0.0001	10.149	5.191	19.845	**< 0.0001**
IPF	3.221	1.207	8.598	0.0195	1.423	0.522	3.882	0.4903
GERD	1.287	0.963	1.720	0.0876				
Obesity	0.168	0.024	1.193	0.0745				
Cancer	2.409	2.090	2.776	< 0.0001	2.006	1.735	2.319	**< 0.0001**
Cirrhosis	2.160	1.441	3.237	0.0002	1.275	0.847	1.920	0.2440
Congestive heart failure	3.124	2.268	4.302	< 0.0001	1.749	1.262	2.426	**0.0008**
Stroke	1.337	0.789	2.265	0.2806				
Diabetes mellitus	1.929	1.647	2.259	< 0.0001	1.221	1.037	1.438	**0.0166**
SLE	3.311	2.022	5.423	< 0.0001	6.323	3.808	10.500	**< 0.0001**
RA	2.087	1.208	3.606	0.0084	2.305	1.318	4.034	**0.0034**
Dermatomyositis	2.213	0.312	15.681	0.4267				
Transplant, others^a^	2.107	0.790	5.622	0.1364				

Abbreviation: *BMI* Body mass index, *CKD* Chronic kidney disease, *COPD* Chronic obstructive pulmonary disease, *eGFR* Estimated glomerular filtration rate, *GERD* Gastroesophageal reflux disease, *IPF* Idiopathic pulmonary fibrosis, *RA* Rheumatoid arthritis, *SLE* Systemic lupus erythematosus

^a^Other than kidney transplant

The p value data in multivariate analysis are bold if < 0.05

In multivariate Cox regression (Table 3), we adjusted all factors found to be significant in the univariate analysis, and the final model showed that old age (HR: 4.439 [3.539–5.583] for age > 65 years old; HR: 2.040 [1.592–2.614] for age 55–65 years old; HR: 1.392 [1.079–2.797] for age 40–55 years old, compared with age < 40 years old), male (HR: 2.558 [1.977–2.578], and poor kidney function since CKD stage 3b were correlated with higher TB risk. In patients with deteriorated kidney function, the risk of TB development increased significantly from CKD stage 3b to stage 5, similarly to the results of univariate analysis but with the risk adjusted downward. The HR of CKD stage 3a was 1.215 (95% C.I.: 0.995–1.484, *p* = 0.0563), HR: 1.538 in stage 3b ([1.182–2.002], *p* = 0.0014), 2.065 in stage 4 ([1.473–2.894]), and 2.877 in stage 5 ([1.872–4.421]) in comparison with stages 1 and 2. The risk in patients with long-term dialysis was HR of 2.041 (1.092–3.815, *p* = 0.0254), which is similar to that of stage 4 CKD. The HR was 15.269 in those with kidney transplant, and it was much higher than those of other subgroups. The proportion of ever corticosteroid use (*n* = 24 [63.2%] vs *n* = 87,922 [30.4%], *p* < 0.001) and immune-suppressants use (*n* = 21 [55.3%] vs *n* = 2239 [0.8%], *p* < 0.001) during the follow up were higher in patients receiving kidney transplant than other patients. In addition, the underlying diseases of COPD, bronchiectasis, pneumoconiosis, cancer, congestive heart failure, diabetes mellitus, SLE and RA were independent conditions for favoring TB development. Among them, pneumoconiosis (HR: 10.149), SLE (HR: 6.323), bronchiectasis (HR: 2.959), RA (HR: 2.305), and cancer (HR: 2.006) were the most important (HR >2.0) clinical diseases predisposing TB development.

## Discussion

In the present study, patients with decline in kidney function of CKD stage 3 and worse had increasing incidences and HR for TB development. The incidence and HR of TB in CKD stage 5 seemed around two-fold and 1.4-fold, respectively, relative to those with long-term dialysis although the 95% C.I.s were overlapped. Decline in kidney function is assumed to influence immune function [10] and is associated with oxidative stress and inflammation due to the decrease of renal clearance of toxins [13]. In addition, Vitamin D insufficiency is not uncommon among patients with CKD or dialysis and might lead to immune dysfunction [19]. Many immune cells such as T cells and B cells, as well as natural killer cells, will be attenuated when renal function has deteriorated [20–22]. Previously, many studies focus on the TB risk in dialysis population [23, 24], but few studies report increasing TB incidence in patients with CKD [14, 25]. Cheng et al. reported the adjusted HR of pulmonary TB was 1.45-fold higher in the CKD group than in the non-CKD group. The effect of TB susceptibility due to differences in kidney function has scarcely been reported before [13]. Cho et al. analyzed a community-based cohort and showed that stage 1–4 CKD had a 25% increase in TB hazard than those without CKD [25]. The present study was conducted based on a large hospital cohort and report the association between TB risk and different stages of impaired kidney function.

The incidence and HR of developing active TB rise significantly from CKD stage 3 to stage 5. This finding is compatible with a previous assumption [13] that the decline of immunity begins and wastes accumulate significantly in stage 3 CKD. The risk of TB worsens in advanced stages of CKD and is highest at CKD stage 5 (incidence of 349.29 per 100,000 person-years), where it is around 7.0-fold that of the 55–65 year-old general population [26]. But the rate of increase in the adjusted HR begins to slow from stage 4 to stage 5. However, the unadjusted HR within stage 5 slightly declines as eGFR decreases (Fig. 2).

For patients receiving long-term dialysis, the TB incidence is similar to that of patients with CKD stage 3, and the adjusted HR resembles that of patients with stage 4 CKD (175.6 per 100,000 person-years; adjusted HR: 2.041). The possible explanation is that the dialysis remove partial uremic toxin and might decrease the uremia related immune suppression [27]. Therefore, the TB risk of advanced CKD might be much higher than that of our previous consideration. Because the WHO suggests active LTBI screening in patients with dialysis, those with stage 3 CKD or higher should also be targeted for TB prevention [6, 8]. Such screening needs to be incorporated into a CKD care bundle to counter their higher infection risk. On the other hand, patients with kidney transplants still have very high risk of TB development. The incidence of 1429.33 per 100,000 person-years is around 48-fold that of the same-aged general population [26]. This result is much higher than those in a previous report [28] and might have been influenced by the small case number in this cohort. It might also be due to the higher proportion of corticosteroid and immune-suppressants use that may be correlated with increasing TB incidence [29]. However, the influences by corticosteroid and immunosuppressants need to be analyzed in further study specially designed for drug dose, duration and equivalent effect.

In addition to the stage of kidney impairment, TB development was also significantly associated with old age, male gender, and underlying co-morbidities such as underlying airway and immunocompromised diseases in the multivariate Cox regression analysis. They are all well known risk factors for TB [30–33] and might play a role in developing TB. In the future, we might integrate the risk factors to validate this model and form a prediction score model.

There are several limitations in this study. First, this was a hospital-based study design. Although we only enrolled patients who were regularly followed up at the study hospital, there was a risk of outcome underestimation bias. In addition, the retrospective design might have missed some data in the record review, such as TB contact history and BCG vaccination. Third, because the study hospital is a tertiary referral center, selection bias existed and TB incidence as well as underlying co-morbidities might have been higher than those in the general population.

## Conclusions

Patients with impaired kidney function, eGFR ≤60 ml/min/1.73m^2^, had increasing risk for TB development. The risk reached 2.877-fold and 2.041-fold HR of TB risk in patients with stage 5 CKD and in those with long-term dialysis, respectively, in comparison with those with CKD ≤ stages 2. The results indicate that patients with advanced CKD are susceptible to TB and have a higher risk than patients receiving dialysis. In the development of LTBI prevention strategies for patients with long-term dialysis, those with advanced CKD should also be considered.

## Supplementary information

**Additional file 1 Table S1.** Demographics of study population according to development of tuberculosis (TB). **Table S2.** Incidence rates of tuberculosis (TB) if we excluded patients lost to follow up < 30 days. **Table S3.** Incidence rates of tuberculosis (TB) if we excluded patients lost to follow up < 180 days.

## Data Availability

Not applicable.

## Abbreviations

CKD: Chronic kidney disease
eGFR: Estimated glomerular filtration rate
HR: Hazard ratio
LTBI: Latent tuberculosis infection
MDRD: Modification of Diet in Renal Disease
TB: Tuberculosis
WHO: World Health Organization
