# Supplementary material for: The impact on incident tuberculosis by kidney function impairment status: analysis of severity relationship
Source: Respir Res. 2020 Feb 12;21:51. doi: 10.1186/s12931-020-1294-5 (PMC7017479; doi:10.1186/s12931-020-1294-5)
Supplement: Supplementary file 1 — Additional file 1 Table S1. Demographics of study population according to development of tuberculosis (TB). Table S2. Incidence rates of tuberculosis (TB) if we excluded patients lost to follow up < 30 days. Table S3. Incidence rates of tuberculosis (TB) if we excluded patients lost to follow up < 180 days. [file 12931_2020_1294_MOESM1_ESM.docx]

**Additional File**

Original Article title

**The impact on incident tuberculosis by kidney function impairment status: analysis of severity relationship**

Chin-Chung Shu^1,2^, Chung-Yu Chen^2,3^, Yi-Chun Yeh^4^, Hsien-Ho Lin^5^, Yu-Feng Wei^6^, Ping-Huai Wang^7^, Shih-Lung Cheng^7^, Jann-Yuan Wang^1,2^, Chong-Jen Yu^1,2^

**Institutions:**

^1^Department of Internal Medicine, National Taiwan University Hospital, Taipei, Taiwan

^2^College of Medicine, National Taiwan University, Taipei, Taiwan

^3^Department of Internal Medicine, National Taiwan University Hospital Yunlin Branch, Yunlin County, Taiwan

^4^Department of Medical Research, National Taiwan University Hospital

^5^College of Public Health, National Taiwan University, Taipei, Taiwan

^6^Department of Internal Medicine, E-Da Hospital, Kaohsiung city, Kaohsiung, Taiwan

^7^Department of Internal Medicine, Far-Eastern Memorial Hospital, New Taipei city, Taiwan

**Corresponding author**:

Chung-Yu Chen, MD, PhD.

Division of Pulmonary and Critical Care Medicine, Department of Internal Medicine, National Taiwan University Hospital Yunlin Branch

No.579, Sec. 2, Yunlin Rd., Douliu City, Yunlin County 640, Taiwan (R.O.C.)

Tel: 886-5-5323911-5675

Fax: 886-5-5335373

E-mail: [c8101147@ms16.hinet.net](mailto:c8101147@ms16.hinet.net)

**Running Title:** TB risk in patients with different kidney functions

Table S1. Demographics of study population according to development of tuberculosis (TB)

| Variables | TB  (n = 1012) | Non-TB  (n = 288567) | *P*-value |
| --- | --- | --- | --- |
| Age (years) | 64.6 ± 16.5 | 51.7 ± 17.3 | < 0.0001 |
| Age group |  |  | < 0.0001 |
| ≤ 40 | 95 (9.4) | 81578 (28.3) |  |
| 40–55 | 162 (16.0) | 78335 (27.2) |  |
| 55–65 | 202 (20.0) | 59649 (20.7) |  |
| > 65 | 553 (54.6) | 69005 (23.9) |  |
| Male | 670 (66.2) | 130475 (45.2) | < 0.0001 |
| eGFR | 80.7 ± 34.3 | 87.3 ± 25.9 | < 0.0001 |
| Kidney function status |  |  | < 0.0001 |
| CKD nil or stage 1 | 362 (35.8) | 129059 (44.7) |  |
| CKD stage 2 | 395 (39.0) | 127853 (44.3) |  |
| CKD stage 3a | 120 (11.9) | 17108 (5.9) |  |
| CKD stage 3b | 64 (6.3) | 7052 (2.4) |  |
| CKD stage 4 | 37 (3.7) | 3412 (1.2) |  |
| CKD stage 5 | 22 (2.2) | 2478 (0.9) |  |
| Long-term dialysis | 10 (1.0) | 1569 (0.5) |  |
| Kidney transplant | 2 (0.2) | 36 (0.0) |  |
| COPD | 43 (4.3) | 3105 (1.1) | < 0.0001 |
| Asthma | 19 (1.9) | 3335 (1.2) | 0.0322 |
| Bronchiectasis^†^ | 14 (1.4) | 884 (0.3) | < 0.0001 |
| Pneumoconiosis^†^ | 9 (0.9) | 96 (0.0) | < 0.0001 |
| IPF^†^ | 4 (0.4) | 414 (0.1) | 0.0604 |
| GERD | 48 (4.7) | 10192 (3.5) | 0.0373 |
| Obesity | 1 (0.1) | 1650 (0.6) | 0.0461 |
| Cancer | 255 (25.2) | 36520 (12.7) | < 0.0001 |
| Cirrhosis | 24 (2.4) | 3651 (1.3) | 0.0017 |
| Congestive heart failure | 39 (3.9) | 4052 (1.4) | < 0.0001 |
| Stroke | 14 (1.4) | 2974 (1.0) | 0.2676 |
| Diabetes mellitus | 190 (18.8) | 29386 (10.2) | < 0.0001 |
| SLE^†^ | 16 (1.6) | 1299 (0.5) | < 0.0001 |
| RA | 13 (1.3) | 1643 (0.6) | 0.0026 |
| Polymyositis^†^ | 0 (0.0) | 106 (0.0) | 1.0000 |
| Dermatomyositis^†^ | 1 (0.1) | 123 (0.0) | 0.3522 |
| Transplant, others^†^* | 4 (0.4) | 488 (0.2) | 0.0956 |

**Abbreviation:** CKD, chronic kidney disease; COPD, chronic obstructive pulmonary disease; eGFR, estimated glomerular filtration rate; GERD, gastroesophageal reflux disease; IPF, idiopathic pulmonary fibrosis; RA, rheumatoid arthritis; SLE, systemic lupus erythematosus

The number means mean ± standard deviation and number (%)

Chi-square tests for categorical variables; t tests for continuous variables.

^†^ Comparison by Fisher’s exact test.

*Other than kidney transplant.

Table S2. Incidence rates of tuberculosis (TB) (N = 313043, TB event = 1203) if we excluded patients lost to follow up < 30 days.

| Variable | HR | 95% CI | | *P*-value | TB events | N | Follow up (years) | Incidence rate  (per 100000 person-years) |
| --- | --- | --- | --- | --- | --- | --- | --- | --- |
| Kidney function |  |  |  |  |  |  |  |  |
| CKD nil or stage 1 | Reference |  |  |  | 430 | 139673 | 518239.18 | 82.97 |
| CKD stage 2 | 1.033 | 0.906 | 1.178 | 0.6231 | 468 | 138197 | 557235.84 | 83.99 |
| CKD stage 3a | 2.355 | 1.945 | 2.851 | < 0.0001 | 139 | 18825 | 71816.29 | 193.55 |
| CKD stage 3b | 3.340 | 2.624 | 4.252 | < 0.0001 | 78 | 7877 | 28037.44 | 278.20 |
| CKD stage 4 | 4.120 | 3.011 | 5.637 | < 0.0001 | 43 | 3941 | 12204.90 | 352.32 |
| CKD stage 5 | 4.643 | 3.146 | 6.852 | < 0.0001 | 27 | 2735 | 6333.05 | 426.33 |
| Long-term dialysis | 3.295 | 2.001 | 5.428 | < 0.0001 | 16 | 1755 | 5720.55 | 279.69 |
| Kidney transplant | 17.045 | 4.249 | 68.371 | < 0.0001 | 2 | 40 | 140.16 | 1426.98 |

**Abbreviation:** CKD, chronic kidney disease

Table S3. Incidence rates of tuberculosis (TB) (N = 272946, TB event = 884) if we excluded patients lost to follow up < 180 days.

| Variable | HR | 95% CI | | *P*-value | TB events | N | Follow up (years) | Incidence rate  (per 100000 person-years) |
| --- | --- | --- | --- | --- | --- | --- | --- | --- |
| Kidney function |  |  |  |  |  |  |  |  |
| CKD nil or stage 1 | Reference |  |  |  | 322 | 122282 | 514168.67 | 62.63 |
| CKD stage 2 | 0.998 | 0.858 | 1.162 | 0.9827 | 346 | 121002 | 553181.36 | 62.55 |
| CKD stage 3a | 2.351 | 1.886 | 2.931 | < 0.0001 | 105 | 16102 | 71163.57 | 147.55 |
| CKD stage 3b | 3.051 | 2.281 | 4.080 | < 0.0001 | 53 | 6589 | 27723.28 | 191.18 |
| CKD stage 4 | 4.129 | 2.856 | 5.969 | < 0.0001 | 31 | 3149 | 12025.58 | 257.78 |
| CKD stage 5 | 4.473 | 2.746 | 7.286 | < 0.0001 | 17 | 2334 | 6243.15 | 272.30 |
| Long-term dialysis | 2.557 | 1.318 | 4.959 | 0.0055 | 9 | 1454 | 5647.34 | 159.37 |
| Kidney transplant | 11.618 | 1.634 | 82.596 | 0.0143 | 1 | 34 | 138.47 | 722.17 |

**Abbreviation:** CKD, chronic kidney disease
